# CuInTe_2_ Nanocrystals: Shape and Size Control, Formation Mechanism and Application, and Use as Photovoltaics

**DOI:** 10.3390/nano9030409

**Published:** 2019-03-11

**Authors:** Guanwei Jia, Baokun Liu, Kun Wang, Chengduo Wang, Peixu Yang, Jinhui Liu, Weidong Zhang, Rongbin Li, Shaojun Zhang, Jiang Du

**Affiliations:** 1 Henan Province Industrial Technology Research Institute of Resources and Materials, Zhengzhou University, Zhengzhou 450001, China; jiaguanwei@126.com (G.J.); 13673387324@163.com (B.L.); 13592589651@163.com (K.W.); wangcd@zzu.edu.cn (C.W.); yangpx@zzu.edu.cn (P.Y.); jhliu13s@alum.imr.ac.cn (J.L.); zhangwd@zzu.edu.cn (W.Z.); zhangshaojun@zzu.edu.cn (S.Z.); 2 School of Physics and Electronics, Henan University, Kaifeng 475004, China; 3 School of metallurgical and Ecological Engineering, University of Science and Technology Beijing, Beijing 100083, China; lirongbin822@163.com; 4 Department of Chemical Engineering, Texas Materials Institute, Center for Nano- and Molecular Science and Technology, The University of Texas at Austin, Austin, TX 78712, USA

**Keywords:** CuInTe_2_, formation mechanism, nanocrystals, nanorods, photovoltaics

## Abstract

We report on the synthesis of CuInTe_2_ nanoparticles and their function in photovoltaic equipment, such as solar cells. Under certain synthesis conditions, the CuInTe_2_ nanocrystals form shape with nanocrystals, nanorods or nanocubes. It was found that CuTe nanocrystals could be converted to CuInTe_2_ by addition of an In reactant. CuInTe_2_ nanorods were synthesized using this method.

## 1. Introduction

I–III–VI_2_ semiconductors have proved to be one of the highest power conversion efficiency photovoltaic materials in thin film photovoltaic applications [1,2,3,4]. In particular, cells with efficiency exceeding 20% has been produced using the Cu(In,Ga)Se_2_ as a solar absorber layer [5].

Copper indium telluride (CuInTe_2_) is mainly used as an important I–III–VI_2_ semiconductor, which has application in thermoelectric [6], photoluminescence [7], nanowire [8,9,10], and photovoltaic devices [11,12,13,14,15]. The direct bandgap of CuInTe_2_ is between 0.91 and 1.02 eV [16] at around 27 °C, which is slightly narrower in comparison to CuInSe_2_ thin film (1.04 eV) [17]. The narrow bandgap absorber (Eg < 1 eV) is required for making use of the bottom cells for multi-junction (tandem) solar cells. Meanwhile, CuInTe_2_ single-junction photovoltaic devices (PVs) have been made with power conversion efficiency (PCEs) of up to 5.1% [18]. Compared to CuInS_2_ and CuInSe_2_, CuInTe_2_ provides a more significant quantum confinement effect and a greater Bohr radius, with the virtue of the covalent property of tellurium [19].

The record efficiency PV materials of Cu(In, Ga)Se_2_ [5], and CuInTe_2_ [18] are fabricated by co-evaporation, which leads to a sharp increase of the costs. Cu(In,Ga)Se (CIGS) has been fabricated as a nanoparticle dispersion solar coating that can be printed or sprayed. Therefore, it could, ideally, omit the procurement of intensive postdeposition [20]. Solar paints could adopt large and continuous roll-to-roll technology on almost all types of surfaces with moderate conditions. The CuInTe_2_ nanocrystals can be obtained either with microwave irradiation [21,22], by solvothermal synthesis [23], or by using a silicate matrix method [19]. However, by using these methods, the CuInTe_2_ was heavily aggregated and could not be well dispersed and; thus, could not be used for solar paints.

We demonstrate the potential of stearic acid to govern the fabrication of CuInTe_2_ nanocrystals and detail a facile synthetic approach for CuInTe_2_ nanocrystals. The CuInTe_2_ nanocrystals are used for PVs and show preliminary efficiency. We further demonstrate the growth mechanism of CuInTe_2_ nanocrystals and evolve it into a general method to directly convert CuTe into CuInTe_2_.

Except for the size control of CuInTe_2_ nanoparticles, stearic acid can also improve the dispersity of CuInTe_2_ nanoparticles in polar solvent and; thus, can be used for solar paints. The cation exchange routes give us a new synthetic method to synthesis CuInTe_2_ with a nanorod morphology, which has not been reported previously.

## 2. Materials and Methods

### 2.1. Materials

Tellurium powder (99.99%), copper(II) acetylacetonate (Cu(acac)_2_) (99.99+%), CuCl (99.99+%), Indium(III) acetylacetonate (In(acac)_3_) (99.99+%), stearic acid (98.5+%), trioctylphosphine (TOP) (97%), 1-octadecene (ODE) (90%), and CdSO_4_ (99.999%) were received from Aldrich Chemical Co. (Milwaukee, United States); Oleylamine (OLA) from TCI America (Portland, United States); ethanol (absolute), toluene (99.99%), and ammonium hydroxide (18 M NH_3_; ACS certified) from Fisher Scientific (Waltham, United States); and thiourea (>99.0%) from Sigma-Aldrich (St. Louis, United States). Oleylamine was degassed overnight at 110 °C under vacuum. All other chemicals were used without additional purification.

### 2.2. CuInTe_2_ Nanocrystals (2 mmol stearic acid) Synthesis

In a typical synthesis, 0.5 mmol of Cu(acac)_2_ and 0.5 mmol of In(acac)_3_ were mixed with 2 mmol stearic acid and 12 mL of ODE in a 25 mL three-neck flask. The mixture was heated under vacuum to 110 °C to obtain a clear blue solution and kept at this temperature for 30 min to remove low-boiling-point impurities. 0.5 mmol OLA was injected into the flask and the mixture was degassed for a further 30 min. The temperature was then increased, under nitrogen, to 170 °C and 1.0 mL of 1 M TOP-Te was injected into the flask. Upon injection, the solution color immediately changed to dark brown. Just after injection, the temperature was set to 230 °C, and CuInTe_2_ nanocrystals were allowed to grow for 20 min. After cooling to room temperature, centrifugation was used to wash the particles using ethanol and toluene as antisolvent and solvent, respectively. Toluene was added to reach a final nanocrystal concentration of 20 mg/mL.

### 2.3. CuInTe_2_ Nanocrystals (4 mmol stearic acid) Synthesis

The same procedures were carried out to get larger size CuInTe_2_ nanocrystals, but stearic acid was increased from 2 to 4 mmol.

### 2.4. CuTe Nanocubes Synthesis

1.0 mmol of CuCl was mixed with 12 mL of OLA in a 25 mL flask. The mixture was heated under vacuum to 110 °C to obtain a clear solution and kept at this temperature for 30 min to remove low-boiling-point impurities. The temperature was then increased, under nitrogen, to 180 °C and 1.0 mL of 1 M TOP-Te was injected into the flask. Upon injection, the solution color immediately changed to deep green. CuTe nanocubes were allowed to grow for 60 min. After cooling to room temperature, centrifugation was used to wash the particles using ethanol and toluene as antisolvent and solvent, respectively. Toluene was added to reach a final nanocrystal concentration of 20 mg/mL.

### 2.5. CuTe Nanorods Synthesis

The same procedures were carried out to get the CuTe nanorods, but the TOP-Te injection temperature was lowered to 90 °C.

### 2.6. Conversion of CuTe to CuInTe_2_

0.5 mmol (0.096g) of CuTe (without taking the mass fraction of ligands into account) and 0.5 mmol of In(acac)_3_ were mixed with 4 mmol of stearic acid and 12 mL of ODE in a 25 mL three-neck flask. The mixture was heated under vacuum to 110 °C and kept at this temperature for 30 min to remove low-boiling-point impurities. 0.5 mmol OLA was injected into the flask and the mixture was degassed for a further 30 min. The temperature was then increased, under nitrogen, to 170 °C and 0.5 mL of 1 M TOP-Te was injected into the flask. The temperature was set to 230 °C, and the convert process was allowed for 20 min. After cooling to room temperature, centrifugation was used to wash the particles using ethanol and toluene as antisolvent and solvent, respectively. Toluene was added to reach a final nanocrystal concentration of 20 mg/mL.

### 2.7. Materials Characterization

Current−potential (IV) characteristics were collected using a Keithley 2400 source meter under AM 1.5G illumination (100 mW/cm^2^). The National Institute of Standards and Technology (NIST) calibrated Si photodiode (Hamamatsu, S1787−08) was used to tune light intensity. External quantum efficiency (EQE) was measured as previously described [24]. Monochromatic light (Newport Cornerstone 260 1/4M) at wavelengths ranging from 300 to 1300 nm, in 10 nm steps, was chopped at 213 Hz and focused to a 1-mm diameter spot size on the device at zero bias. EQE was measured using a lock-inamplifier (Stanford Research Systems, model SR830) after calibrating light intensity with silicon (Hamamatsu) and germanium (Judson) photodiodes. 

Low-resolution transmission electron microscopy (TEM) images were acquired on a FEI Tecnai Spirit Bio Twin operated at 80 kV. High-resolution transmission microscopy (HRTEM) images were acquired on a field emission JEOL 2010F TEM operated at 200 kV. The JEOL 2010F TEM is equipped with an Oxford INCA EDS detector, which was used to collect EDS data. UV−vis−NIR absorbance spectra were acquired with a Varian Cary 500 UV−vis−NIR spectrophotometer.

X-ray diffraction (XRD) was performed using a Rigaku R-Axis Spider diffractometer with an image-plate detector and Cu Kα (λ = 1.54 Å) radiation operated at 40 kV and 40 mA, respectively. XRD samples were prepared by drying a drop of concentrated nanoparticle dispersion onto a glass slide in a glovebox. The nanocrystal powder was then suspended on a 0.5 mm nylon loop using mineral oil for analysis. Samples were scanned for 10 min while rotating at 5°/s. The 2D diffraction patterns were integrated using the Rigaku 2DP powder processing suite, with subtraction of the background scattering from the nylon loop and mineral oil.

Raman spectroscopy was performed using a 514 nm Ar ion laser source operated at 0.5 mW using a Renishaw microscope system set up in reflection mode. The beam was focused through a 50x objective, making a spot size approximately 1.3 µm in diameter on the sample.

### 2.8. Device Fabrication

CuInTe_2_ nanocrystal PVs were fabricated with an Au/CuInTe_2_/CdS/i-ZnO/indium tin oxide (ITO) device structure. A 5 nm layer of Cr followed by 60 nm of Au were thermally deposited onto soda lime glass (Delta Technologies, 25 × 25 × 1.1 mm polished float glass). Films of CuInTe_2_ nanocrystals were spray deposited from toluene at room temperature. A CdS buffer layer was deposited by dropping 0.7 mL of a CdS precursor solution (1.25 mL of 15 mM CdSO_4_, 2.2 mL of 1.5 M thiourea, and 2.8 mL of 18 M NH_4_OH in water) onto the CuInTe_2_ nanocrystal film, heated to 95 °C on a hot plate, and covered with an inverted crystallization dish for 2 min. The substrate was removed from the hot plate, rinsed with deionization (DI) water, and dried with a stream of compressed air. Top layers of i-ZnO and ITO were deposited by radio frequency (RF) sputtering from a ZnO target (Lesker, 99.9%) in a 0.5% O_2_ in Ar atmosphere (Praxair, 99.95%) and an ITO target (Lesker, 99.99% In_2_O_3_:SnO_2_ 90:10) in Ar atmosphere (Praxair, research grade). ZnO and ITO were deposited selectively onto 8 rectangular regions with active device areas of 0.08 cm^2^. Silver paint was applied for electrical contact to the devices.

## 3. Results

**Figure 1:**
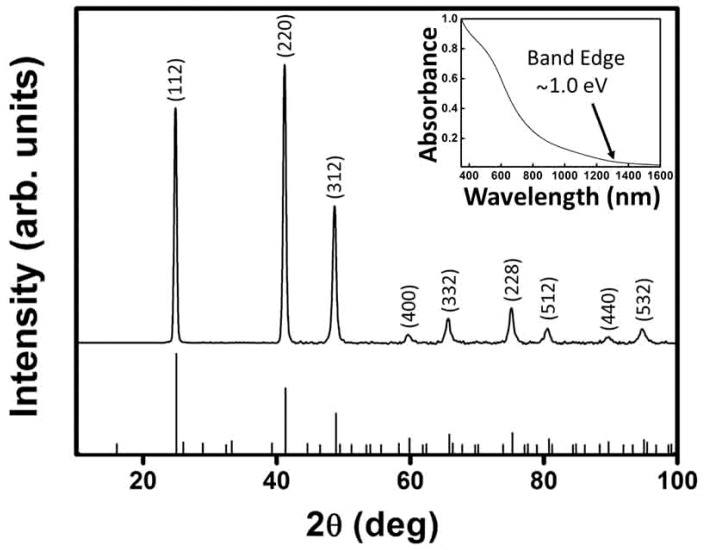
X-ray diffraction (XRD) of CuInTe_2_ nanocrystals (2 mmol stearic acid). The peak labels correspond to those of sphalerite (cubic) CuInTe_2_ (PDF No. 43-1401). Inset: CuInTe_2_ nanoparticles are dispersed in toluene and their UV-vis-near infrared absorbance spectrum is demonstrated.

The nanocrystals were confirmed as sphalerite (cubic) CuInTe_2_ through X-ray diffraction (XRD), which are shown in Figure 1. EDS from fields of the nanoparticles showed the mean Cu/In/Te composition of 0.24:0.28:0.48, which was close to the ideal target 0.25:0.25:0.50 ratio. The band gap energy was 1.0 eV through the optical absorbance spectra of nanocrystals dispersion. And the 1.0 eV was near to the reference value 1.02 eV for CuInTe_2_ [6].

**Figure 2:**
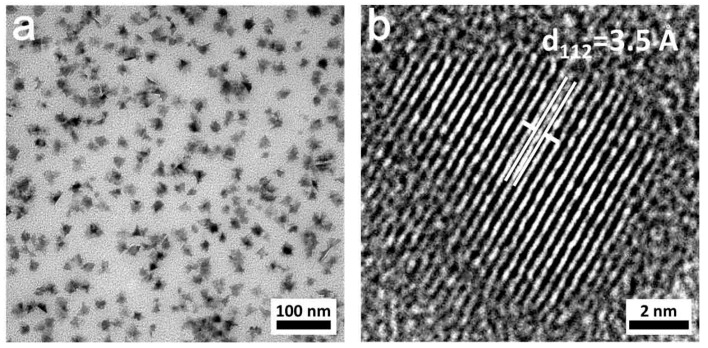
Transmission electron microscopy (TEM) (**a**) and high-resolution transmission microscopy (HRTEM) (**b**) of CuInTe_2_ nanocrystals (2 mmol stearic acid).

TEM images of CuInTe_2_ nanocrystals are revealed in Figure 2. The mean diameter of nanocrystals was 15.3 ± 3.6 nm, which were crystalline. Many of these CuInTe_2_ nanocrystals had sharp edges, which was also observed on the surface of co-evaporated CuInTe_2_ films [18]. Compared to co-evaporated CuInSe_2_ films, under the same co-evaporated conditions, CuInTe_2_ film had larger and more faceted surface [25], which relate to the physical properties of a relatively low epitaxy reaction temperature (347 °C) and melting point (789 °C).

**Figure 3:**
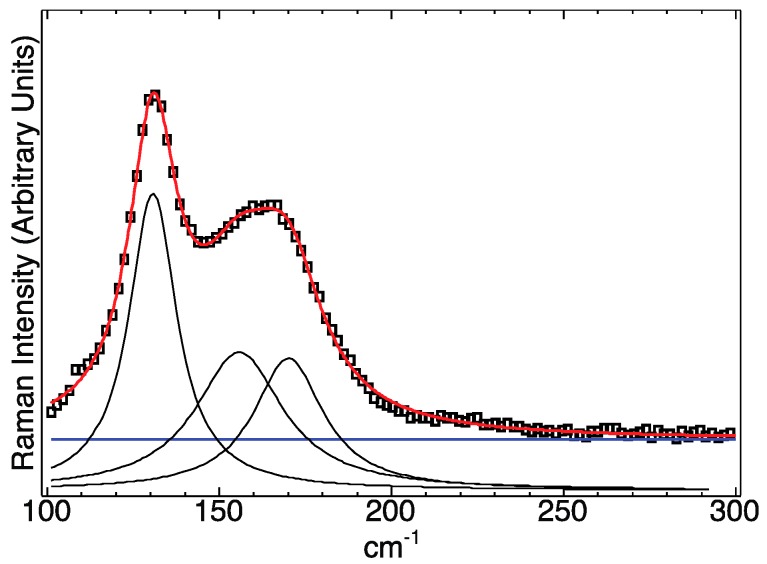
Deconvoluted CuInTe_2_ spectrum shows peaks at 131, 156, and 170 cm^−1^. Black squares are actual data, and the red line is the sum of all the fitted components. The blue horizontal line is a fitted parameter to account for the background caused by ligand fluorescence.

As shown in Figure 3, peaks were deconvoluted using Lorentzians. The most prominent of these peaks appeared at 131 cm^−1^, which we assigned to the A_1_ mode of CuInTe_2_. This A_1_ mode shifted to higher wavenumbers compared to the previously calculated and observed value (127 cm^−1^). [26] Similar shifts of the A_1_ mode have been previously studied in CuInSe_2_ and indicate a lack of chalcopyrite cation ordering [27,28]. In sphalerite CuInSe_2_, such a shift of the A_1_ mode to higher wavenumbers was accompanied by the disappearance of certain XRD peaks that are characteristic of the chalcopyrite structure. Those missing peaks all have odd integers for *l* in the Miller index (*hkl*) and, if present, would confirm chalcopyrite cation ordering. From Figure 3, it can be seen that the nanocrystals used in this investigation lacked diffraction peaks containing odd values of *l*. Due to the shift of the A_1_ mode to higher wavenumbers and the lack of diffraction peaks containing odd *l*-values, the shalerite structure was confirmed.

Furthermore, the deconvoluted Raman peaks at 156 cm^−1^ and 170 cm^−1^ were in good agreement with previously observed peaks (158.5 cm^−1^ and 169.9 cm^−1^) [26], and match fairly well to the theoretically estimated E^2^ (160.8 cm^−1^) and E^3^ (171.5 cm^−1^) CuInTe_2_ modes [29] based on a simplified Keating model. Full Lorentzian fit parameters are found in the Table S1.

**Figure 4:**
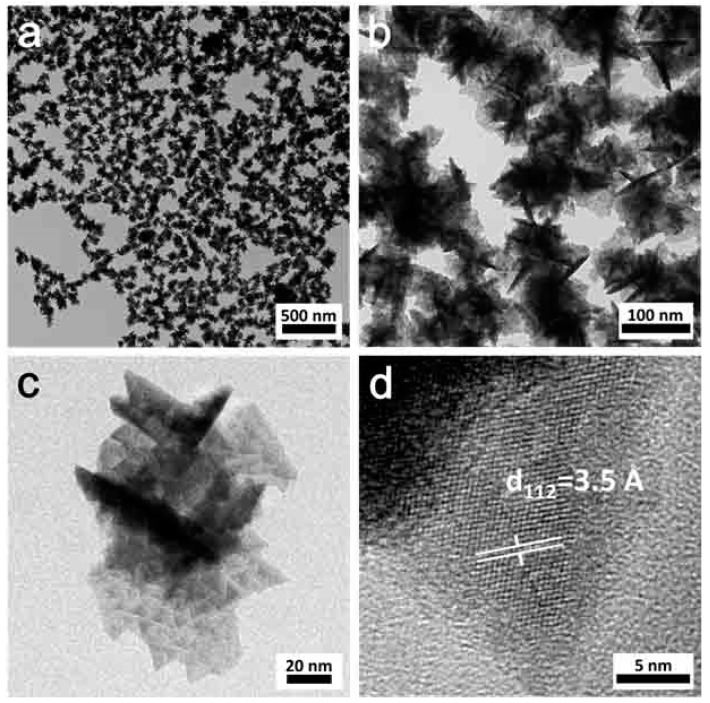
TEM (**a**–**c**) and HRTEM (**d**) of CuInTe_2_ nanocrystals (4 mmol stearic acid).

For the CuInTe_2_ nanocrystals, the size and morphology can be governed through adjusting the stearic acid/metal ratio. Figure 4 demonstrates the CuInTe_2_ about the morphology of nanocrystals (4 mmol stearic acid), obtained by increasing the stearic acid–metal ratio from 4:1 to 8:1. The CuInTe_2_ nanocrystals (4 mmol stearic acid) were crystalline (Figure 4d) and had triangle-shaped edges and bodies (Figure 4c). XRD (Figure S1) confirmed that the nanocrystals (4 mmol stearic acid) were sphalerite (cubic) CuInTe_2_. We assumed that stearic acid concentration dominated nanocrystals nucleation. When the concentration of stearic acid was increased, a lower nucleation rate was initially obtained, increasing the total amount of precursor for posterior nanocrystals growth. Consequently, these larger CuInTe_2_ nanocrystals (4 mmol stearic acid) were ultimately obtained. Meanwhile, compared to CuInSe_2_ nanoparticles under similar synthetic conditions [20], the morphology of CuInTe_2_ nanoparticles were more faceted. Similar results have been reported in the synthesis of CuInTe_2_ and CuInSe_2_ thin films by the co-evaporation system under the same growth conditions. This notable morphology feature of CuInTe_2_-based thin films relates to the physical properties of a relatively low epitaxy temperature (347 °C) [30] and melting point (789 °C) [31] compared to CuInSe_2_. Models for the different crystal growth mechanisms, symmetrical polyhedrons, are presented elsewhere [32].

**Figure 5:**
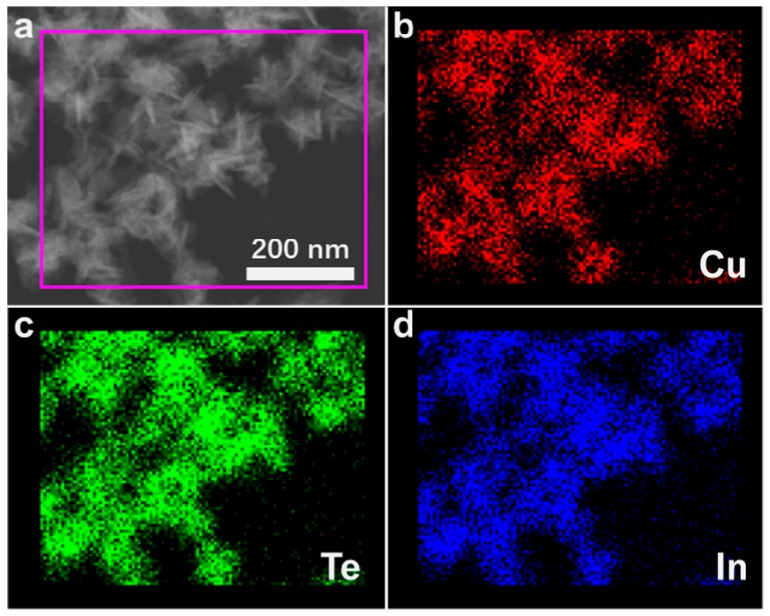
Scanning transmission electron microscopy (STEM) and energy-dispersive X-ray (EDX) elemental mapping of Cu, In, and Te of CuInTe_2_ nanocrystals (4 mmol stearic acid).

The composition of the CuInTe_2_ nanocrystals (4 mmol stearic acid) was ascertained by the elemental maps from energy-dispersive X-ray (EDX) spectroscopy (Figure 5). EDS from the sample of the CuInTe_2_ nanocrystals (4 mmol stearic acid) revealed a mean Cu–In–Te containment of 0.24:0.26:0.50.

**Figure 6:**
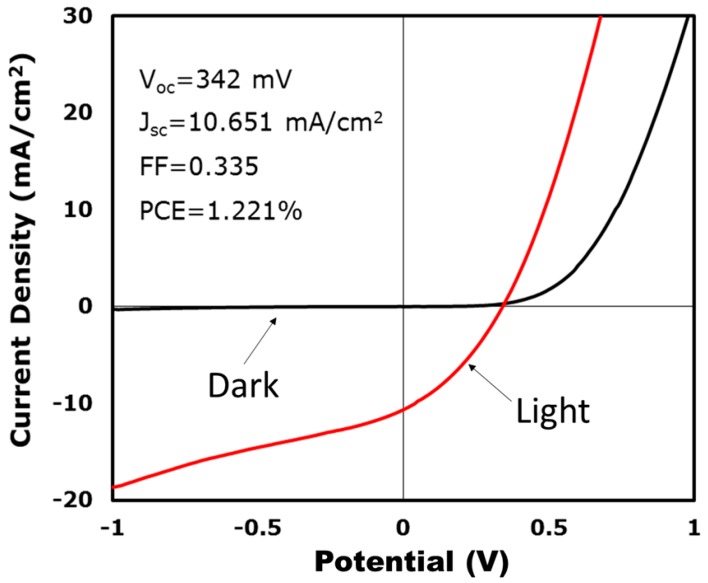
Current–voltage properties of a CuInTe_2_ nanocrystals photovoltaic (PV) device.

Photovoltaic devices were fabricated by using CuInTe_2_ nanocrystals as the absorber layer (2 mmol stearic acid). Similar to CIGS, CuInTe_2_ coatings were typically p-type [33], and test equipment was composed of a layered structure that consisted of Au/CuInTe_2_/CdS/ZnO/indium tin oxide (ITO). CuInTe_2_ nanocrystals were deposited by spray coating with a toluene dispersion. The anneal was unnecessary for the nanocrystal layer. Figure 6 (in Table S2 and Figure S6) displays the PV response of a typical device having an open-circuit voltage (VOC) of 342 mV, a short-circuit current density (JSC) of 10.651 mA/cm^2^, a fill factor (FF) of 0.335, and a power conversion efficiency (PCE) of 1.221% with the conditions AM 1.5. Compared with the previous report [12], the photovoltaic performance parameters (Voc, Jsc, and FF) were lower. The reason was that the absorber layer was not annealed or chemically treated.

**Figure 7:**
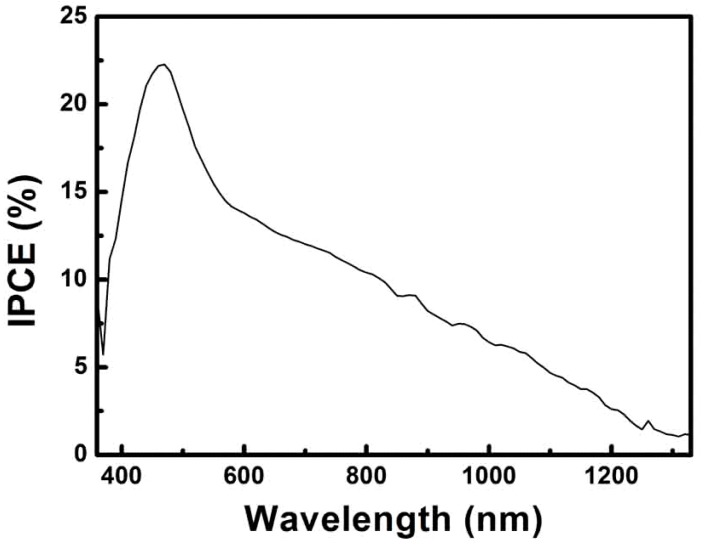
Incident photon conversion efficiency (IPCE) spectra of the CuInTe_2_ nanocrystals PV device.

The incident photon conversion efficiency (IPCE) (Figure 7) was in accordance with the absorbance spectra of the CuInTe_2_ nanoparticles. Additionally, the response results were ascertained by the CuInTe_2_ nanoparticles. The relatively high IPCE of ∼22.5%, for wavelengths between 400 and 500 nm, tailed off at higher wavelengths. The strong photovoltaic response of IPCE in the 400–500 nm region might have been due to the cadmium sulfide (CdS) layer [34,35,36]. The long wavelength IPCE cutoff at ∼1250 nm corresponded to the optical gap (1.02 eV) of the CuInTe_2_ nanoparticles, and the sharp drop in IPCE at wavelengths <400 nm was the result of ZnO light absorption [20]. 

**Figure 8:**
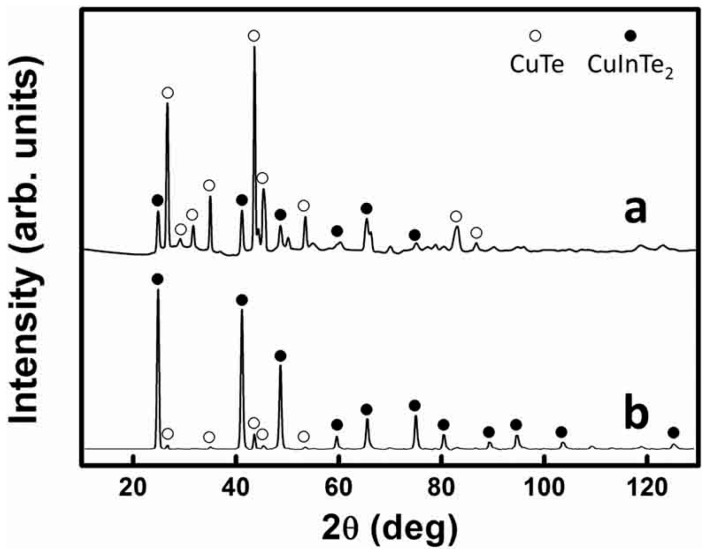
XRD of when the CuInTe_2_ nanocrystals reaction was taken: (**a**) 5 min; and (**b**) 10 min. (CuTe: portable document format (PDF) No. 26-0524 and CuInTe_2_: PDF No. 43-1401).

**Figure 9:**
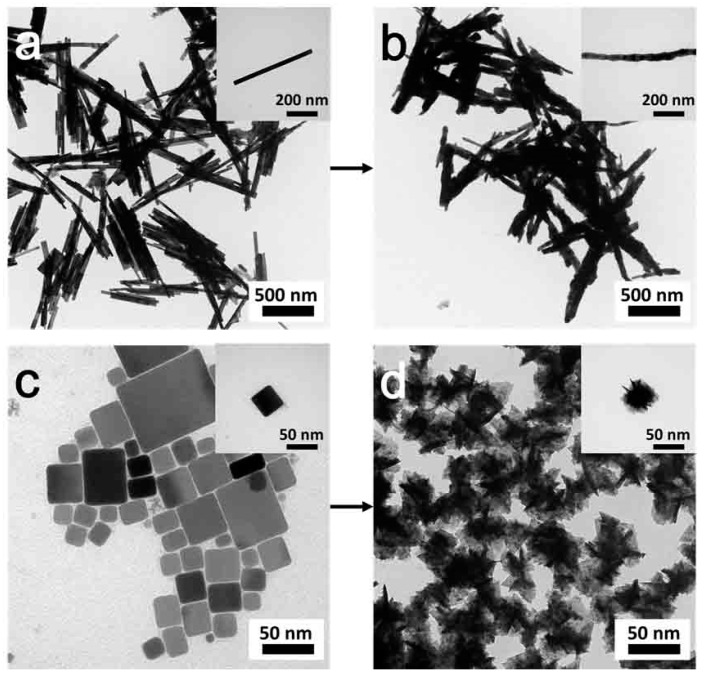
TEM of converting (**a**) CuTe nanorods into (**b**) CuInTe_2_ nanorods, and converting (**c**) CuTe nanocubes into (**d**) CuInTe_2_ nanocrystals.

Time-chasing XRD studies of CuInTe_2_ nanocrystals were carried out to obtain information on the growth mechanism of this system. XRD (Figure 8a) showed that when the reaction time was 5 min, CuTe nanocrystals formed as a major phase together with minor CuInTe_2_. The reason for CuTe forming as the major phase, formed in the initial stage of the reaction, may be due to the greater reactivity of the Cu-aliphatic amines complex, compared with In-aliphatic amines complexes [37]. After 10 min of the reaction, CuInTe_2_ ends up being favored (Figure 8b). However, it is interesting to hypothesize CuTe being converted into CuInTe_2_ as a transition according to TEM images shown in the Figure 9.

Following this growth mechanism hypothesis, we successfully directly converted CuTe nanorods (TEM in Figure 9a, XRD data in Figure S2a) and CuTe nanocubes (TEM in Figure 9c, XRD data in Figure S2c) into CuInTe_2_ nanorods (TEM in Figure 9b, XRD data in Figure S2b, HR-TEM in Figure S5a) and CuInTe_2_ nanocubes (TEM in Figure 9d, XRD data in Figure S2d, HR-TEM in Figure S5b) under the same reaction conditions (Figure S2). CuTe nanorods and nanocubes can be synthesized by varying the TOP-Te injection temperature in the OLA system (Figure 9a,c). XRD and HRTEM (Figures S2 and S5) proved the crystal phase, composition, and lattice structure of CuInTe_2_. Time-chasing XRD (Figures S3 and S4) of the converting process also confirmed that, after the reaction at 5 min, CuInTe_2_ started forming. It should be a facile and general method to directly convert CuTe (with different morphologies) into CuInTe_2_.

**Figure 10:**
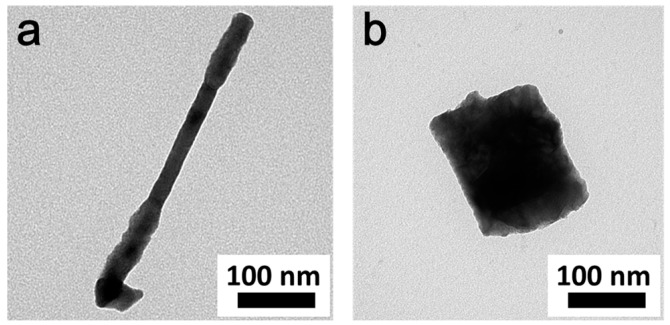
TEM of converting (**a**) CuTe nanorods and (**b**) CuTe nanocubes into CuInTe_2_. The reaction time was 5 min.

As per the TEM (Figure 10) shown, it was the hypothesis that when the convert reaction time was 5 min, the converting process of the CuInTe_2_ started at the two ends of the CuTe nanorods and at the surface of the CuTe nanocubes.

## 4. Conclusions

In conclusion, a synthetic approach for obtaining CuInTe_2_ nanocrystals with different morphologies has been extended. The size morphology of CuInTe_2_ nanocrystals can be governed through adjusting the stearic acid–metal ratio. PV equipment assembled with the synthetic CuInTe_2_ nanocrystals demonstrated PCEs of increments up to 1.221% (PCEs of all devices on the chip are shown in Table S2 and Figure S6). However, owing to the lack of the process of deposition, with any high-temperature or chemical means on the solar absorber layers, the power conversion efficiency of the devices was slightly lower. In order to improve the PV efficiency for application, the synthetic and device fabrication process should be further optimized. The formation mechanism of CuInTe_2_ nanocrystals was explored and we invented a facile and general method to directly convert CuTe nanostructures into CuInTe_2_. CuInTe_2_ nanorods were firstly synthesized, and it could be used for advanced nanodevices such as sensors and photodetectors, etc.

## Supplementary Materials

The following are available online at https://www.mdpi.com/2079-4991/9/3/409/s1, Figure S1: XRD of (a) CuInTe_2_ nanocrystals (4 mmol stearic acid). Figure S2: XRD of converting (a) CuTe nanorods into (b) CuInTe_2_ nanorods, and (c) CuTe nanocubes into (d) CuInTe_2_ nanocrystals. Figure S3: XRD of converting CuTe nanocubes into CuInTe_2_ nanocrystals. The reaction time was 5 min. Figure S4: XRD of converting CuTe nanorods into CuInTe_2_ nanorods. The reaction time was 5 min. Figure S5: HRTEM of (a) CuInTe_2_ nanorods and (b) CuInTe_2_ nanocrystals. (Converted from CuTe nanorods and CuTe nanocubes). Figure S6: Current–voltage properties of a CuInTe_2_ nanocrystals PV device. Table S1: Fitted Lorentzian values for Raman data. Table S2: PCEs and *I-V* curves of all devices on the chip.
