# Supplementary material for: CuInTe2 Nanocrystals: Shape and Size Control, Formation Mechanism and Application, and Use as Photovoltaics
Source: Nanomaterials (Basel). 2019 Mar 11;9(3):409. doi: 10.3390/nano9030409 (PMC6474138; doi:10.3390/nano9030409)
Supplement: Supplementary file 1 [file nanomaterials-09-00409-s001.pdf]

$CuInTe_2$ Nanocrystals: Shape and Size Control, Formation Mechanism and Application, and Use as Photovoltaics

Guanwei Jia [1,2], Baokun Liu [1], Kun Wang [1], Chengduo Wang [1], Peixu Yang [1], Jinhui Liu [1], Weidong Zhang [1], Rongbin Li [3], Shaojun Zhang [1] and Jiang Du [1,4,*]

1 Henan Province Industrial Technology Research Institute of Resources and Materials, Zhengzhou University, Zhengzhou 450001, China; 13673387324@163.com (B.L.); 13592589651@163.com (K.W.); wangcd@zzu.edu.cn (C.W); yangpx@zzu.edu.cn (P.Y.); jhliu13s@alum.imr.ac.cn (J.L.); zhangwd@zzu.edu.cn (W.Z.); zhangshaojun@zzu.edu.cn (S.Z.)
2 School of Physics and Electronics, Henan University, Kaifeng 475004, China; jiaguanwei@126.com (G.J.)
3 School of metallurgical and Ecological Engineering, University of Science and Technology Beijing, Beijing 100083, China; lirongbin822@163.com
4 Department of Chemical Engineering, Texas Materials Institute, Center for Nano- and Molecular Science and Technology, The University of Texas at Austin, Austin, Texas 78712, USA
* Correspondence: 0210927@163.com

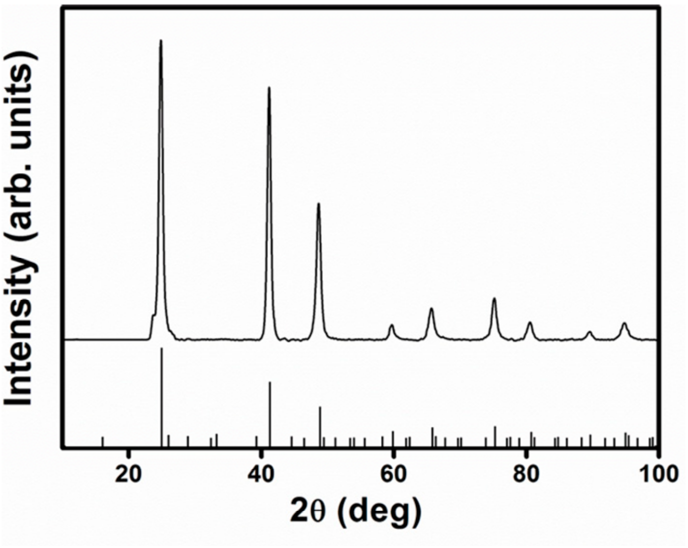

Figure S1. XRD of (a) $CuInTe_2$ nanocrystals (4 mmol stearic acid) (PDF No. 43-1401)

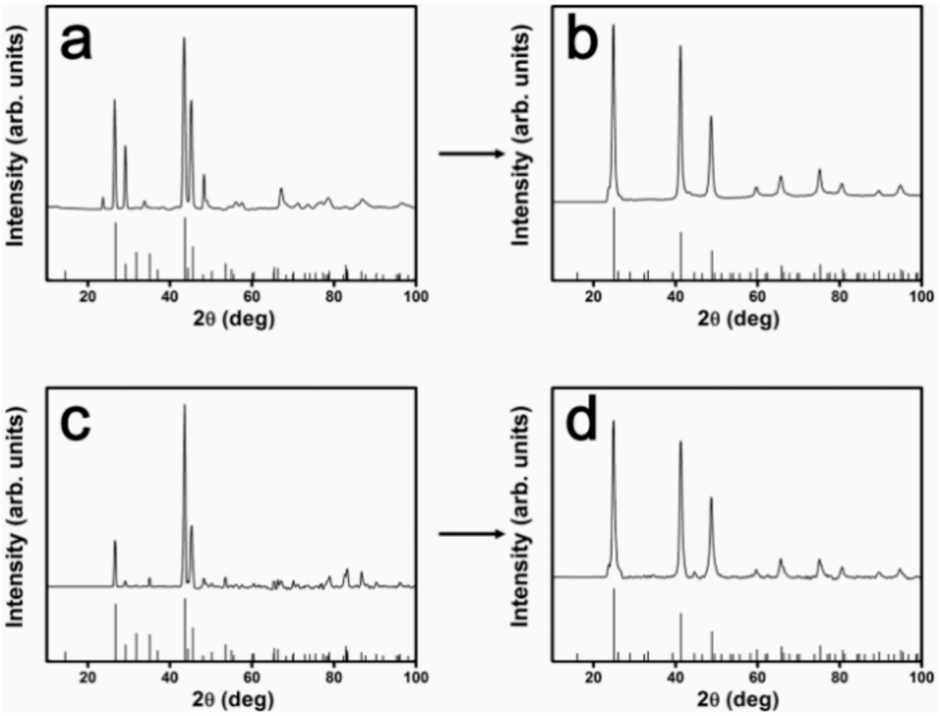

Figure S2. XRD of converting (a) CuTe nanorods into (b) $CuInTe_2$ nanorods and (c) CuTe nanocubes into (d) $CuInTe_2$ nanocrystals (CuTe :PDF No. 26-0524)

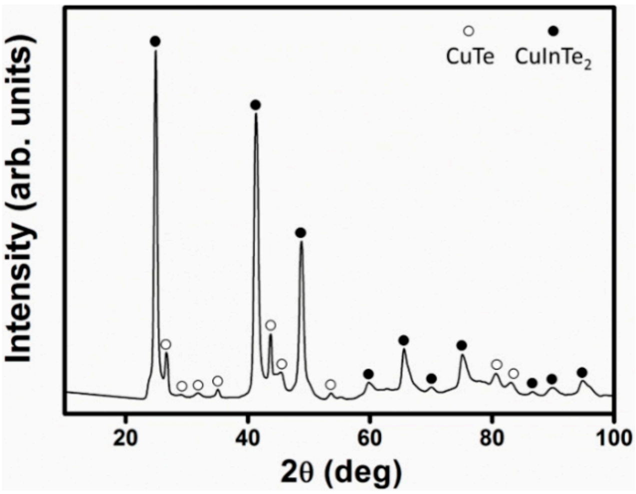

Figure S3 XRD of converting CuTe nanocubes into $CuInTe_2$ nanocrystals. The reaction time was 5 min.

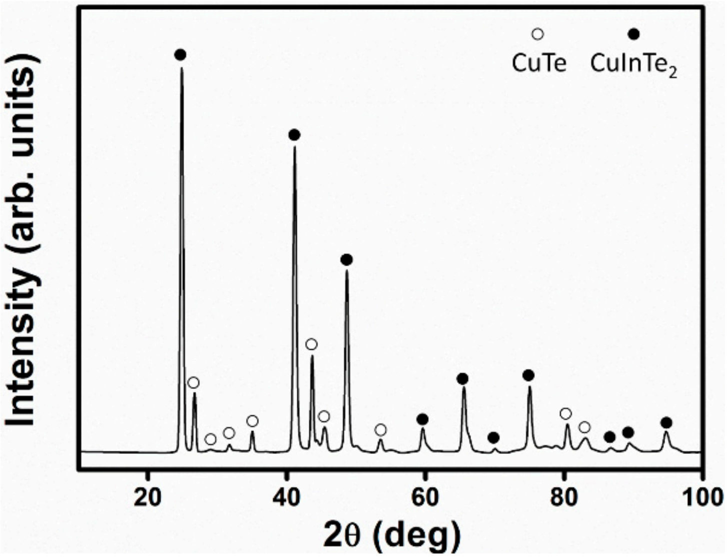

Figure S4. XRD of converting CuTe nanorods into $CuInTe_2$ nanorods. The reaction time was 5 min.

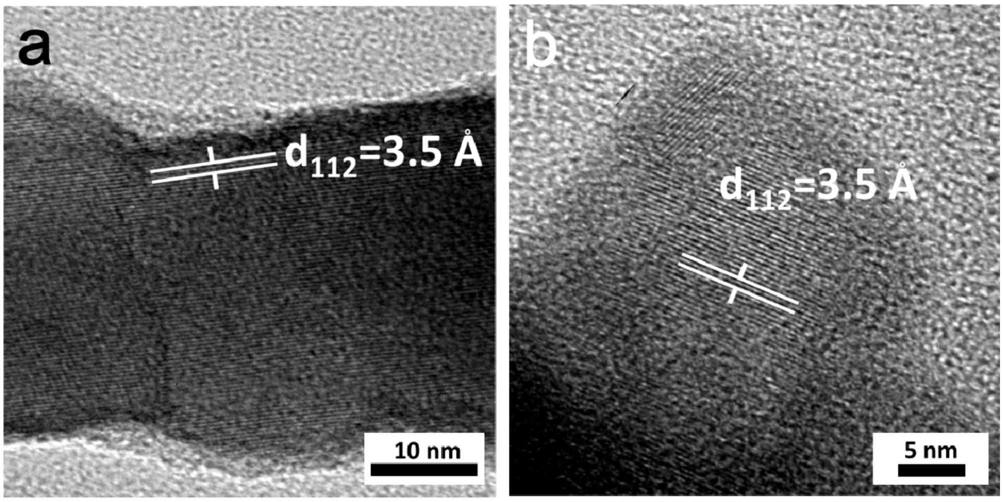

Figure S5. HR-TEM of (a) $CuInTe_2$ nanorods (Figure 9(b)) and (b) $CuInTe_2$ nanocrystals (Figure 9(d). (Converted from CuTe nanorods and CuTe nanocubes)

Raman peak values were obtained using Lorentzian fits to Raman data. First, the spectrum was normalized so that the largest feature in the 100-300 cm^{-1} range would equal an intensity value of 100. Table 2 shows the fitted peaks as well as the fitted fluorescent baseline parameter.

Table S1. Fitted Lorentzian values for Raman Data

	Peak or Value	FWHM	Area
Fitted Baseline	12.931 (arbitrary intensity)	-	-
Peak 1	130.68 cm^{-1}	16.849 cm^{-1}	1988.9
Peak 2	155.73 cm^{-1}	30.695 cm^{-1}	1687.7
Peak 3	170.06 cm^{-1}	24.404 cm^{-1}	1281.2

Table S2. PCEs and I-V curves of all devices on the chip

Device No.	PCE (%)	Voc (V)	Jsc (mA/cm^2)	FF
01	1.010	0.354	-9.771	0.292
02	1.136	0.349	-10.123	0.322
03	1.221	0.342	-10.651	0.335
04	1.089	0.357	-9.949	0.307
05	1.069	0.356	-10.284	0.301
06	1.139	0.342	-10.996	0.330
07	1.120	0.348	-10.218	0.315
08	1.008	0.347	-9.955	0.292

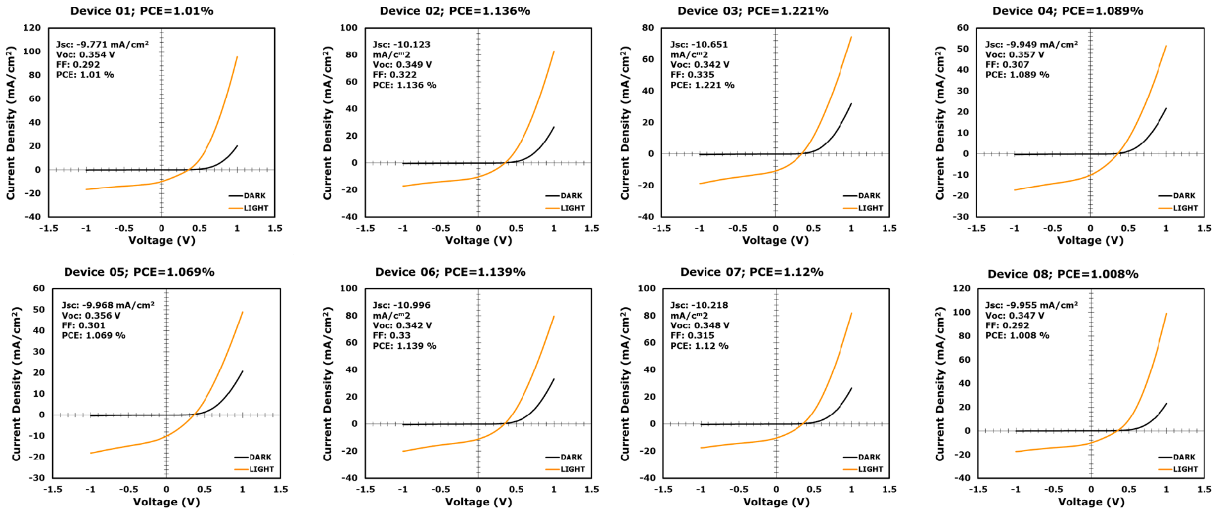

Figure S6 Current-voltage properties of a $CuInTe_2$ nanocrystals PV device
